# The Institutional Worker

**Published:** 1912-01-27

**Authors:** 


### THE INSTITUTIONAL WORKER

**Being a Special Supplement to "The Hospital"**

### Editor's Notices.

**Contributions:**

Contributions should be written, or preferably typed, on one side of the paper only, and all articles sent in are accepted upon the distinct understanding that they are forwarded to The Hospital only.

The Editor cannot undertake to return MSS. not used, but when a stamped directed envelope is enclosed the MSS. may be returned if a special request is made.

Accepted articles and paragraphs of news will be paid for after publication at the scale rate.

**Address:**

To prevent delay all contributions and letters on editorial business must be addressed exclusively to the Editor, "The Hospital," 29 Southampton Street, Strand, London, W.C. It is important that this regulation shall be strictly observed.

**Correspondence:**

Correspondence on all subjects is invited. The name and address of correspondents must be given as a guarantee of good faith, but not necessarily for publication.

**Special Articles:**

Special articles are invited, and questions, inquiries, and paragraphs upon all matters relating to the work, administration, and management of general, special, mental, fever, cottage and convalescent hospitals, sanatoria, homes, institutions, societies and organisations for the treatment or care of the sick, injured and dependents of all classes. Every matter affecting the interests and welfare of the staff of all grades working in these institutions will receive special consideration.

**Administrative Medicine, Research and Items of News:**

Special terms will be paid for approved articles on subjects in this wide field by experts who have made some section of it their special study and interest. We wish to give prominence to every movement tending to advance accuracy of diagnosis, efficiency in treatment, and the development of modern methods to eradicate disease and promote the welfare of the suffering.

**A Bureau of Information:**

We invite inquiries and applications for information and help in providing for that numerous class of sufferers who require special care or house-room which they cannot obtain in their own homes or secure for themselves. We cannot, however, prescribe or recommend practitioners.

**Books for Review:**

Publishers are particularly requested to send advance proofs of any new books of importance whenever possible, as the Editor has made arrangements to publish immediate reviews on a new plan.

**Photographs, Plans, Blocks, and Illustrations:**

It is requested that wherever possible MSS. may be accompanied by illustrations in any of the above forms. The name of the sender and of the article to which it belongs should be written on the back of each photograph, drawing, or block for purposes of identification.

**Local Papers:**

Newspapers containing reports or news paragraphs should be marked and addressed to the Sub-Editor.

**The Coupon System:**

A coupon will be found at the bottom of the third inside page of the cover each week. This coupon must be attached to every question or inquiry to which an answer is desired in The Hospital.

### Manager's Notices.

**To Institutional Workers.**

Your interests are our interests. The Hospital is a national institution, having been for upwards of a quarter of a century the leading exponent of institutional life and work. Its columns are open to you every week. Whatever your needs, The Hospital is undoubtedly one of the finest mediums in the country for advertising your requirements. The prestige of this journal is universally recognised as one of the greatest factors which has contributed to its success. New features and interesting articles have been introduced, and there is abundant testimony from all quarters that The Hospital is highly appreciated for the excellence of its contents. Many commendatory letters have reached us conveying congratulations on the distinct improvement in the journal, and since the reduction in price to one penny the circulation of The Hospital has not only increased considerably, but the paper has extended its sphere of influence, giving ample evidence of its popularity throughout the institutional world.

**Institution Authorities.**

The economical management of an Institution depends very greatly upon purchasing supplies in the cheapest market.

It is often impossible locally to secure the highest quality at the lowest prices.

In consequence, a section will be reserved in this part of the paper for announcements of Institutions inviting Tenders for Contracts.

This will enable Authorities of Institutions to secure tenders for supplies from all over the country, and ensure purchases at the lowest possible prices consistent with quality.

The charge for Tenders is 6s. per inch, and for this small outlay many pounds may be saved on supplies of all descriptions.

Special Rates will be quoted for those institutions which agree to send all their vacancy, contract, and public notices to The Hospital each year, so as to make it a file of such announcements for ready reference.

**Classified Advertisements:**

All small advertisements will be classified, for this section of the paper is widely read and of special interest. We wish to encourage those wanting appointments who are attracted to institutional work, and therefore offer them the reduced rate of *twenty-one words and under for* 1s., and *for every ten and part of ten words beyond*, 6*d*.

This form may be used for small prepaid advertisements, and when filled up should be posted to the

*Manager*, The Hospital,
28 and 29 Southampton Street,
Strand, W.C.

| | |
|---|---|
| Appointments Wanted, 21 words | ... 1s. 0d. |
| Appointments Vacant, 21 words | ... 2s. 6d. |

### OFFICIAL APPOINTMENTS VACANT.

### Poor-Law Vacancies.

Aged Women's Attendant (£24), Bethnal Green Guardians. Matron of the Workhouse, Waterloo Road, Victoria Park, N.E.

Assistant Case-Paper Clerk (£2 weekly), Islington Guardians. Mr. E. Davey, C. to G., St. John's Road, Upper Holloway, N. Jan. 29.

Assistant Female Attendant (£16), St. Pancras Guardians. Mr. J. E. P. Hall, C. to G., Town Hall, Pancras Road, N.W.

Assistant Female Cook (£24), Bristol Guardians. Mr. J. J. Simpson, C. to G., Bristol. Jan. 27.

Assistant Labour Master (£28), York Union. Mr. G. Sykes, C. to G., York. Jan. 30.

Assistant Nurse (£20), Lewes Union. Mr. C. Patrick, C. to G., Lewes. Feb. 1.

Assistant Nurse (£26), Andover Union. Mr. W. R. Graham, C. to G., Andover. Feb. 1.

Assistant Nurse (£25), Bromsgrove Union. D. H. Holloway, C. to G., Union Offices, Bromsgrove.

Assistant Nurses (£25), Grimsby Union. Mr. J. F. Wintringham, C. to G., Grimsby. Feb. 1.

Barber and Bathman (£25), Rotherham Union. Mr. W. C. Harrison, C. to G., Rotherham. Jan. 27.

Caretaker and Gardener and Cook (£30 and £20), Rotherham Union. Mr. W. C. Harrison, C. to G., Rotherham. Jan. 27.

Charge and Midwifery Nurse (£32), Northampton Union. W. Fawkes, C. to G., 4 Derngate, Northampton. Jan. 27.

Charge Nurse (£35), Thornbury Union. H. P. Thurston, C. to G., Union Offices, Thornbury, Glos. Feb. 1.

Charge Nurse (£30), Birkenhead Union. J. Carter, C. to G., Poor Law Offices, Birkenhead. Jan. 30.

Charge Nurses (£30), City of London Union. Matron of the Infirmary, Clifden Road, Median Road, N.E.

Clerk to Stoke Board of Guardians and Assessment Committee (£350 about). Mr. H. G. Poole, Acting C. to G., Stoke-on-Trent. Jan. 30.

Cook (£26), Steyning Union. Mr. A. Flowers, C. to G., Shoreham-by-Sea. Jan. 29.

Cook (£24), Castle Ward Union. Mr. C. S. Shortt, C. to G., 18 Bigg Market, Newcastle-on-Tyne. Jan. 29.

Cook (£30), Farnham and Hartley Wintney Schools. Superintendent of the Schools, Crondall, Hants.

Cook-General (£25), Eastbourne Union. The Matron of the Workhouse, Eastbourne.

Female Assistant Relieving Officer and Infant Protection Visitor (£50), Sevenoaks Union. F. H. Vibert, C. to G., Bank Chambers, Sevenoaks. Feb. 14.

Female Attendant (£20), Tendring Union. Matron, Tendring Infirmary, Weeley, R.S.O.

Female Cook (£30), Brighton Guardians. Mr. B. Burfield, C. to G., Brighton. Jan. 31.

First Assistant Nurse (£26), Walsingham Union. Mr. R. S. Butcher, C. to G., Fakenham. Jan. 27.

Fifth Labour Master (£25), Marylebone Guardians. Master of the Workhouse, Northumberland Street, W.

Foster-Mother (£25), York Union. Mr. G. Sykes, C. to G., York. Jan. 27.

Foster-Mother (£40), Bucklow Union. The C. to G., Knutsford. Feb. 2.

Foster-Mother (£25), Nuneaton Union. Mr. C. Blakeway, C. to G., Nuneaton. Feb. 5.

Foster-Mothers (4) (£28 each), Brentford Union. Mr. W. Stephens, C. to G., Isleworth, W. Jan. 30.

General Assistant (£20), Royston Union. Mr. A. Sharpe, C. to G., Royston, Herts. Jan. 30.

Head Male Attendant (£35), Bristol Guardians. Mr. J. J. Simpson, C. to G., Bristol. Jan. 27.

Head Nurses (3) (£25), Holborn Union. Matron of the Infirmary, Archway Road, N.

Infirmary Matron (£150), Birmingham Guardians. Mr. C. Fletcher, C. to G., Birmingham. Jan. 29.

Labour Master (£25), Tendring Union. Mr. H. J. Burden, Master of the Workhouse, Weeley, Essex.

Laundress (£22), Royston Union. Mr. A. Sharpe, C. to G., Royston, Herts. Jan. 30.

Laundrymaid (£16), St. Pancras Guardians. Mr. J. E. P. Hall, C. to G., Town Hall, Pancras Road, N.W.

Laundrymaid (£16), St. George's Guardians. Matron of the Infirmary, Fulham Road, S.W.

Male Attendant (£30), Fulham Guardians. Mr. E. J. Mott, C. to G., 129 Fulham Palace Road, W. Jan. 29.

Male Attendant (£30), York Union. Mr. G. Sykes, C. to G., York. Jan. 30.

Male Attendant (£25), Grimsby Union. Mr. J. F. Wintringham, C. to G., Grimsby. Feb. 1.

Male Attendant (£30), Aston Union. Mr. J. North, C. to G., Vauxhall Road, Birmingham. Feb. 5.

